# Functional lymphatic reserve capacity is depressed in patients with a Fontan circulation

**DOI:** 10.14814/phy2.14862

**Published:** 2021-05-31

**Authors:** Sheyanth Mohanakumar, Benjamin Kelly, Aida Luiza Ribeiro Turquetto, Mathias Alstrup, Luciana Patrick Amato, Milena Schiezari Ru Barnabe, João Bruno Dias Silveira, Fernando Amaral, Paulo Henrique Manso, Marcelo Biscegli Jatene, Vibeke Elisabeth Hjortdal

**Affiliations:** ^1^ Department of Cardiothoracic and Vascular Surgery Aarhus University Hospital Aarhus Denmark; ^2^ Department of Radiology Aarhus University Hospital Aarhus Denmark; ^3^ Department of Clinical Medicine Aarhus University Aarhus Denmark; ^4^ Department of Cardiothoracic Surgery Rigshospitalet Copenhagen Denmark; ^5^ Heart Institute (InCor) University of Sao Paulo Medical School São Paulo Brazil; ^6^ Ribeirão Preto Medical School – University of São Paulo Ribeirão Preto Brazil; ^7^ Pediatric and Adult Congenital Heart Disease Unit Hospital das Clínicas Ribeirão Preto Brazil

**Keywords:** Fontan circulation, lymphatic dysfunction, lymphatic reserve capacity, near‐infrared fluorescence imaging

## Abstract

**Background:**

Lymphatic abnormalities play a role in effusions in individuals with a Fontan circulation. Recent results using near‐infrared fluorescence imaging disclosed an increased contraction frequency of lymphatic vessels in Fontan patients compared to healthy controls. It is proposed that the elevated lymphatic pumping seen in the Fontan patients is necessary to maintain habitual interstitial fluid balance. Hyperthermia has previously been used as a tool for lymphatic stress test. By increasing fluid filtration in the capillary bed, the lymphatic workload and contraction frequency are increased accordingly. Using near‐infrared fluorescence imaging, the lymphatic functional reserve capacity in Fontan patients were explored with a lymphatic stress test.

**Methods:**

Fontan patients (*n* = 33) were compared to a group of 15 healthy individuals of equal age, weight, and gender. The function of the superficial lymphatic vessels in the lower leg during rest and after inducing hyperthermia was investigated, using near‐infrared fluorescence imaging.

**Results:**

Baseline values in the Fontan patients showed a 57% higher contraction frequency compared to the healthy controls (0.4 ± 0.3 min^−1^ vs. 0.3 ± 0.2 min^−1^, *p* = 0.0445). After inducing stress on the lymphatic vessels with hyperthermia the ability to increase contraction frequency was decreased in the Fontan patients compared to the controls (0.6 ± 0.5 min^−1^ vs. 1.2 ± 0.8 min^−1^, *p* = 0.0102).

**Conclusions:**

Fontan patients had a higher lymphatic contraction frequency during normal circumstances. In the Fontan patients, the hyperthermia response is dampened indicating that the functional lymphatic reserve capacity is depressed. This diminished reserve capacity could be part of the explanation as to why some Fontan patients develop late‐onset lymphatic complications.

## Graphical abstract

We report, for the first time, that the functional lymphatic reserve capacity is depressed in patients with a Fontan circulation. Using Near‐Infrared Fluorescence imaging to asses function and morphology of the peripheral lymphatic vessels in patients with subclinical and clinical symptoms of effusions, we have introduced a new simple way to stress the lymphatic vessels to reveal lymphatic functional capacity.

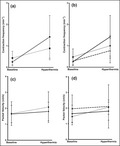

## 1 INTRODUCTION

The Fontan circulation is a palliative surgical option for children born with a univentricular heart allowing them to reach adulthood. Unfortunately, the elevated central venous pressure (CVP) and diminished cardiac output, a physiological consequence of the Fontan circulation, have shown to be injurious on all organ systems (Allen et al., 2017; Dennis et al., 2018). In the recent years, it has been recognized that lymphatic abnormalities and perhaps dysfunction play a role in the pathology in complications seen in patients with a Fontan circulation (Biko et al., 2019; Kelly et al., 2020a; Rychik et al., 2013). Even though increased focus has been on the lymphatic system as a key contributor to the organ deterioration seen in Fontan patients, the pathology behind these complications is still poorly understood (Biko et al., 2019; Dori et al., 2014a; d'Udekem & Leval, 2018; Kreutzer & Kreutzer, 2017; Mohanakumar et al., 2019; Veldtman et al., 2017). Changed lymphatic morphology has been exposed in Fontan patients with lymphatic complications using MRI lymphangiography (Biko et al., 2019; Dori et al., 2014a; Mohanakumar et al., 2019) and it has been demonstrated that these lymphatic abnormalities can be targeted for treatment with percutaneous interventional procedures (Dori, Keller, Fogel, et al., 2014; Savla et al., 2017), However, only one small explorative study has addressed function of the lymphatic vasculature in Fontan patients. Using near‐infrared fluorescence (NIRF) imaging, we showed that the baseline contraction frequency of lymphatic vessels was higher in Fontan patients without complications compared to healthy controls. It is proposed that the elevated lymphatic contraction frequency seen in the Fontan patients is necessary to maintain the same interstitial fluid balance as seen in healthy controls (Mohanakumar et al., 2019). Accordingly, circulating blood level of norepinephrine (NE) has previously been reported to be increased in Fontan patients (Turquetto et al., 2018), a stress hormone that has been shown to increase lymphatic vessel contractility in ex vivo studies (Mohanakumar et al., 2018; Telinius et al., 2010, 2014). But how the lymphatic function is changed in Fontan patients with significant fluid retention is still unknown. We have, in prior studies on healthy subjects, shown that hyperthermia can be used as a stress test to increase lymphatic contraction frequency and indirectly disclose the reserve capacity of the lymphatic vessels ability to contract (Groenlund et al., 2017; Kelly et al., 2020b). We hypothesized that Fontan patients without complications are able to compensate for increased fluid stress by increasing lymphatic function and that the lymphatic function in patients with complications would be impaired even at rest.

Thus, we aimed to describe the functional state and stress response of superficial lymphatics in the lower leg in Fontan patients, using near‐infrared fluorescence (NIRF) imaging, that allow for real‐time visualization of lymph flow (Groenlund et al., 2017; Kelly et al., 2020b; Mohanakumar et al., 2019).

The capillary filtration of fluid (Mohanakumar et al., 2019) and the peripheral blood flow (PBF) were measured using plethysmography (Turquetto et al., 2018). Finally, levels of NE and brain natriuretic peptide (BNP) were measured, the latter for indirect evaluation of the heart function (Niedner et al., 2010).

## 2 METHODS

The data, analytic methods, and study materials will be/have been made available to other researchers for purposes of reproducing the results or replicating the procedure.

### 2.1 Ethical approval

The clinical trial *Lymphatic function and interstitial fluid filtration in patients with a Fontan Circulation* was approved by both Ribeirão Preto‐ (CAAE: 65327917.4.0000.5440) and the InCOR Scientific Committee on Research Ethics, São Paulo, Brazil (CAAE: 88009218.6.0000.0068). The study is registered in clinicaltrials.gov (NCT04394507). Informed written consent was acquired from all study participants before enrolment. The trial was conducted according to the ethical principles for medical research involving human subjects as guided by the Helsinki declaration.

### 2.2 Study population

The inclusion criteria were patients with a Fontan circulation followed at the Pediatric and Adult Congenital Heart Disease Unit, Hospital das Clínicas, Ribeirão Preto and Instituto do Coração (InCor), São Paulo, Brazil. Exclusion criteria included age under 18, mental illness and genetic syndromes. Included patients (*n* = 33) were compared to healthy age‐ and gender‐matched control group (*n* = 15) enrolled through flyers. Patients and controls were included in random order during the study period from July to August 2018.

### 2.3 Study design

The study was a prospective cross‐sectional study, where lymphatic function and capillary fluid filtration were assessed in patients with a Fontan circulation and compared to a group of healthy controls. The study subjects were investigated using NIRF imaging of the lymphatic vessels, strain gauge plethysmography and PBF measurements. PBF and blood samples for measurements of level of NE and BNP were measured at other visits due to logistics. The logistical hindering was also the reason for why not all patients and controls completed blood analysis and PBF measurements.

We defined patients diagnosed with PLE, plastic bronchitis, clinical peripheral edema, previously suffering from unexplained peripheral edema requiring diuretic management and non‐contrast MRI lymphangiography verified thoracic or/and abdominal effusions (all patients underwent MRI lymphangiography as a part of another study), as complicated Fontan patients. The rest of the Fontan patients were considered uncomplicated in terms of struggling with fluid imbalance.

### 2.4 Study protocol

Near‐infrared imaging was used to evaluate the function of the superficial lymphatic vessels in the right lower leg and foot as previously described (Groenlund et al., 2017; Mohanakumar et al., 2019).

The participants were placed in a supine position during NIRF imaging and plethysmography throughout the 4‐h protocol. Briefly, subsequently to 15 min of acclimatizing to room temperature and supine position, the fluorescent dye indocyanine green (ICG) (VERDYE; Diagnostic Green GmbH, Germany) was intradermally injected on three sites on the right foot. The fluorescent dye, 0.1 mL (30 μg ICG), was dissolved in sterile water and diluted with isotonic saline to a final concentration of 0.3 gL^−1^ and injected intradermally with 31G needles (Wiotech, Denmark). ICG taken up by the superficial lymphatic vessels were recorded using an electron‐multiplier charge‐coupled device camera (C9100‐3 Hamamatsu, Japan), with a Navitar lens (25 mm f0.95) with two 835 nm ± 15 nm (>OD5) band‐pass filters mounted in front and behind. Image capture was set to 3.33 S^−1^ and gain was set to 1200. As a light source for excitation of ICG, we used a custom‐designed 785 nm 450 mW laser (PowerTechnology, USA), with a 780 ± 28 nm band‐pass filter and a fixed lens to spread the light. The obtained recordings were displayed in real‐time on a screen and were saved on an external hard drive for post‐analysis (Groenlund et al., 2017; Kelly et al., 2020b; Mohanakumar et al., 2019).

**FIGURE 1:**
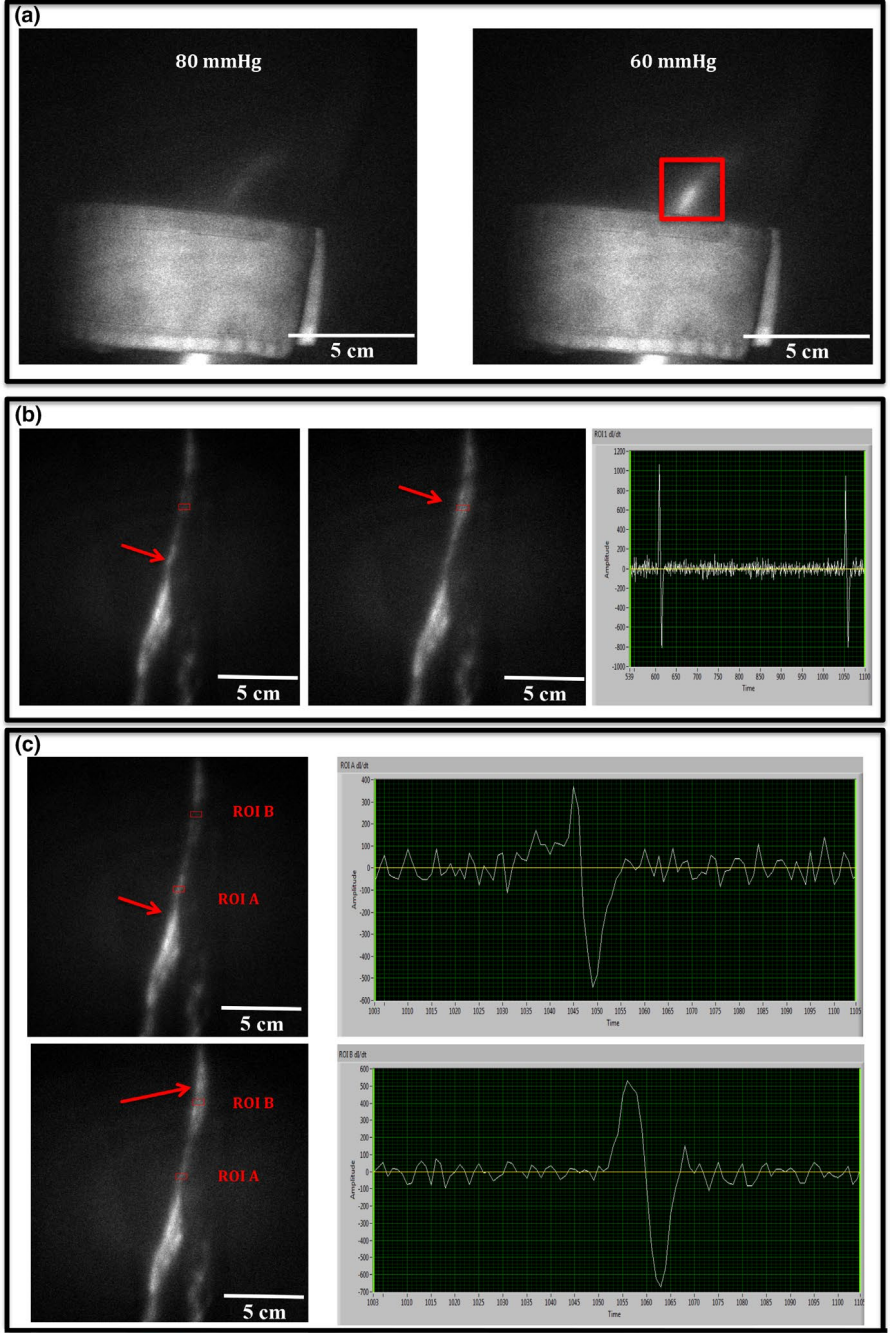
Near‐infrared fluorescence imaging outcomes. (a) Examples of still images from NIRF imaging during pumping pressure measurement of the lower leg; *left* the cuff is inflated to 80 mm Hg and the lymph is accumulated at the distal border of the cuff in the vessel present, while proximal for the cuff the lymphatic vessel is empty, *right* the cuff is slowly deflated and at 60 mm Hg lymph passes under the cuff in the vessel (red box), determining lymphatic pumping pressure. (b) Raw data trace from NIRF imaging and estimation of contraction frequency. *left* A ROI is placed on an empty part of the vessel. Upstream for the ROI a lymphatic package is moving (red arrow). *middle* The lymphatic package has moved into the ROI (red arrow) representing a contraction. *right* Intensity plot from custom‐build LabVIEW lymphatic analysis software. Two spikes are shown in the trace reflecting two contractions computed of an increase followed by a decrease in fluorescence intensity through the ROI. c Raw data trace from NIRF imaging and estimation of package velocity. *upper left* lymphatic package is moving downstream through ROI A (red arrow). *upper right* Raw trace over a time course (sec) for ROI A. *lower left* Lymphatic package have moved 8 cm over 4 s and reached ROI B (red arrow). *lower right* Raw trace over a time course (sec) for ROI B. See Movie S2, https://figshare.com/s/4f1190c22df4a8594175 for baseline sequence. Abbreviations: Near‐infrared fluorescence (NIRF). Region of interest (ROI)

Four sequences were recorded during the NIRF protocol. First, an injections sequence (Movie S1, https://figshare.com/s/4f1190c22df4a8594175). Next, following a 20‐min resting period to allow the lymphatic vessels to accumulate ICG, a 6‐min baseline sequence of the right lower leg was recorded, with the participants lying completely still to eliminate any extrinsic influence on the lymphatic vessels (Movie S2, https://figshare.com/s/4f1190c22df4a8594175). The third sequence was assessment of pumping pressure (Figure 1). Finally, after exposure to hyperthermia a 6‐min sequence was recorded again in a supine position (Movie S2, https://figshare.com/s/4f1190c22df4a8594175).

Lymphatic function was evaluated by measuring; contraction frequency, velocity and pumping pressure. A custom‐written LabVIEW 12.0 program (National Instruments, USA) was used for image analysis. Analyses were conducted blinded and performed twice by two observers (Figure 1).

### 2.5 Baseline contraction frequency

A contraction was defined as a visual validation of a packet moving through a Regions of interests (ROI) placed on each measurable vessel and/or increase in the intensity signal displayed in the LabVIEW program. Contractions were counted over the obtained 6‐min baseline (Figure 1).

### 2.6 Baseline packet velocity

Two ROIs were placed 5–10 cm apart on each vessel in the baseline sequence. Packages moving continuously through both ROIs were included in the calculations. The distance between the two ROIs was divided by the time difference between the packet passing each ROI (Figure 1).

### 2.7 Pumping pressure

Pumping pressure was determined by occluding lymphatic vessels using a Hokanson sphygmomanometer cuff (Marcom Medical Denmark). A tourniquet was placed distally to the sphygmomanometer cuff to prevent any lymphatic flow and allowing manual emptying of the lymphatic vessels under and above the cuff. Subsequently, the cuff was inflated to 80 mm Hg (Hokanson E20 Rapid cuff inflator, Hokanson AG101 air source, SC10 cuff; Marcom Medical, Denmark) and the tourniquet was released allowing lymph flow up to the distal border of the cuff. Then, the cuff pressure was reduced with 5 mm Hg every 5th min until the fluorescent dye passed under the inflated cuff at which point the reached pressure level was noted as the pumping pressure. (Figure 1).

### 2.8 Hyperthermia

To assess lymphatic vessel function after local hyperthermia the right foot and lower leg was submerged in a 40°C heated water‐tank for 5 min and dried without applying pressure to the skin to avoid any massage effect, before acquiring a 6‐min sequence of the same vessels as recorded during the baseline image sequence. The contraction frequency and packet velocity after hyperthermia was estimated as described earlier under baseline (Groenlund et al., 2017; Kelly et al., 2020b).

### 2.9 Capillary filtration rate

Capillary filtration rate (CFR, µL × 100 mL^−1^tissue × min^−1^) was measured using a strain gauge plethysmography setup (Hokanson EC6 and E20; Marcom Medical, Denmark) connected to a PC using an analog‐to‐digital converter (ADInstruments, UK). The measurements were analyzed using Labchart 7 software. A previously well‐described venous congestion protocol was followed. (Mohanakumar et al., 2019) The protocol was slightly modified by adding an extra step increasing the cuff pressure up to 70 mm Hg. The cuff was placed around the right calf before a 6‐step venous congestion protocol was conducted (20–70 mm Hg). After calibration the cuff was rapidly inflated to and 20 mm Hg and held for 3 min before increasing the pressure with 10 mm Hg every 3 min until 70 mm Hg was reached. Distally to the cuff a strain gauge was placed on the widest circumference of the calf and the volume was recorded continuously. Immediately after the acute increase in venous pressure a fast nonlinear increase in volume was observed due to venous‐distension. Eventually, the fast increase reaches steady state and the increase in interstitial fluid volume (edema formation) due to the increased venous pressure and thereby increased capillary hydrostatic pressure, leads to change in CFR and a second phase with a linear slow increase in calf volume. As shown by Stewart et al. The CFR (μL × 100 mL^−1^tissue × min^−1^) was measured as the slope of the time‐volume change (%) curve at steady state at the end of each pressure phase and was measured at each 10 mm Hg pressure step from 20 to 70 mm Hg (Jensen et al., 1985; Mohanakumar et al., 2019; Stewart, 2003).

### 2.10 Peripheral blood flow

Peripheral blood flow (mL/min/100 mL tissue) was evaluated by venous occlusion plethysmography of the right forearm. A mercury‐filled silastic band connected to a low‐pressure transducer and plethysmograph (Hokanson A16), was placed around the forearm 5 cm distally of the elbow. Two cuffs were placed on both sides of the silastic band, on the wrist and the upper arm, respectively. The cuff at the wrist was inflated to overcome arterial pressure (200 mm Hg). After 1 min, the upper arm cuff was inflated to exceed venous pressure (60 mm Hg) continuously in 10 s interims for a duration of 10 s each time. The mercury silastic band was stretched during the 10‐s intervals due to the forearm volume increase and this volume change was recorded (Turquetto et al., 2018).

### 2.11 Plasma concentration of brain natriuretic peptide (BNP) and norepinephrine (NE)

The concentration of NE and BNP was assessed in venous blood samples collected in the morning, after 30 min of rest. Norepinephrine was analyzed by high‐pressure liquid chromatography method with electrochemical detection. The detection limit for norepinephrine at the InCor‐HCFMUSP laboratory is 12.5 pg/mL. with reference values set to 40 to 268 pg/mL (Turquetto et al., 2018).

BNP levels were analyzed with the chemiluminescence immunoassay method with reference value to be less than 100 pg/mL (Niedner et al., 2010).

### 2.12 Statistics

All data were gathered in Research Electronic Data Capture tools hosted at the Institute for Clinical Medicine, Aarhus University. Analyzed data were stored in Microsoft Excel 2019 (16.33) and all statistical analyses and graphical presentation of the data were managed using GraphPad Prism 6 and Stata/SE 15.1. All analyzes and draft of manuscript were prepared at the Department of Cardiothoracic and Vascular Surgery, Aarhus University Hospital.

Data were tested for normality and presented as mean ± standard deviation (SD). Significance was analyzed with the unpaired Student's *t*‐test (data with two samples) and for strain gauge plethysmography data significance was analyzed with two‐way ANOVA. For possible correlation between variables, Pearson's correlation coefficient was used. In all tests, significance level was set to 0.05.

Based on a mean contraction frequency found in a previous NIRF imaging study on Fontan patients of 0.8 min^−1^ and standard deviation of 0.1, inclusion of 33 patients would detect a 10% difference in contraction frequency with a power of 88% and significance level set to *p* < 0.05 (Mohanakumar et al., 2019).

Intraclass correlation (ICC) was estimated to evaluate the intraobserver agreement. The ICC was estimated using the coefficient calculated with a two‐way mixed model for absolute difference between two measurements. The ICC coefficient was presented with 95% confidence intervals.

## 3 RESULTS

### 3.1 Study population

The Fontan patients had various underlying diagnoses (Table 1), all treated with a total cavopulmonary connection (TCPC). Description of the demographical, clinical characteristics and medication can be found in Table 1.

**TABLE 1:** Demographics and clinical characteristics

	Fontan (*n* = 33)	Control (*n* = 15)	*p*‐value
Demographics			
Female (%)	18 (55%)	8 (53%)	0.9394
Age (y)	27 (±7)	27 (±9)	0.8896
BMI	23.5 (±4.1)	23.2 (±4.5)	0.8174
Clinical characteristics			
Blood pressure (mm Hg)	113 (±14)/70 (±14)	120 (±9)/71 (±10)	0.0948/0.6969
Heart rate (beats/min)	79 (±13)	79 (±13)	0.8984
MAP (mm Hg)	87 (±9)	84 (±12)	0.3573
O2 saturation at rest (%)	92 (±5)	98 (±1)	0.0001*
Time with Fontan (years)	15.9 (±6.5)		
Diagnosis, *n*(%)			
Tricuspid valve atresia	16 (48.5%)		
Pulmonary valve atresia	4 (12.1%)		
Mitral atresia	2 (6.1%)		
Double inlet left ventricle	5 (15.1%)		
Others	6 (18.2%)		
Ventricular morphology, *n*(%)			
Left	29 (88%)		
Right	2 (6%)		
Both	2 (6%)		
Complications, *n*(%)			
Portal hypertension	2 (6%)		
Valvular regurgitation	4 (12%)		
Arrythmia	4 (12%)		
AV‐collaterals	4 (12%)		
Pacemaker	0 (0%)		
Medications, *n*(%)			
ACE inhibitor	13 (39%)	0 (0%)	
Antiplatelet	8 (24%)	0 (0%)	
Anticoagulant	23 (69%)	0 (0%)	
Diuretics	7 (21%)	0 (0%)	
Complications with effusions			
PLE	3 (9%)	0 (0%)	
Peripheral edema	4 (12%)	0 (0%)	
Previously edema	8 (24%)	0 (0%)	
MRI lymphangiography verified Effusions	10 (30%)	0 (0%)	

Data are presented as means ± SD.

Abbreviations: BMI indicates body mass index; MAP, mean arterial pressure; PLE, protein‐losing enteropathy

The CVP pressure in the Fontan circulation was not routinely measured so this information was unfortunately not available. The Fontan patients were primarily in New York Heart Association Classification (NYHA) 1 (*n* = 26) (Table 2). Latest echocardiography (*n* = 23) showed adequate systolic function in 18 patients and 5 patients with mild dysfunction. Ejection fraction was estimated to range between 46%–64% (*n* = 12).

**TABLE 2:** Investigational outcomes

	Fontan (*n* = 33)	Control (*n* = 15)	*p*‐value
Biomarkers			
Norepinephrine (pg/mL)	378 (±232)	228 (±82)	0.0464*
BNP (pg/mL)	38 (±39)	14 (±19)	0.0634
Albumin (g/dL, NR: 3.4–5.0)	4.0 (±0.9)		
Bilirubin (mg/dL, NR: <1.0)	0.9 (±0.6)		
ALAT (U/L, NR: 14–59)	41 (±0.2)		
INR (INR, NR: 0.8–1.2)	2.1 (±0.7)		
Clinical characteristics			
Peripheral blood flow (mL/min/100 mL tissue)	1.7 (±0.5)	2.1 (±0.2)	0.0088*
Plethysmography, onset of edema (mm Hg)	42 (±15)	30 (±8)	0.0074*
Echocardiography ‐ Systolic function (*n* = 28), *n* (%)			
Adequate	23 (82%)		
Mild dysfunction	5 (18%)		
Moderate	0 (0%)		
Severe	0 (0%)		
NYHA classification (*n* = 31), *n*(%)			
NYHA 1	26 (84%)		
NYHA 2	4 (13%)		
NYHA 3	1 (3%)		
NYHA 4	0 (0%)		
NIRF imaging morphology, *n* (%)			
Torturous vessels	5 (15%)	1 (6%)	
Dermal backflow	3 (9%)	0 (0%)	

Data are presented as means ± SD.

Abbreviations: ALAT indicates Alanintransaminase; BNP, brain natriuretic peptide; INR, International Normalized Ratio; NIRF, Near‐Infrared fluorescence; NR, Normal range; NYHA, New York Heart Association Classification.

* *p*‐value is statistical significant, *p* < 0.05.

In our study group, we found 16 complicated Fontan patients: diagnosed with PLE (*n* = 3), clinical peripheral edema (*n* = 4), asymptomatic edema patients after treatment with diuretics (*n* = 8), and non‐contrast MRI lymphangiography verified thoracic or/and abdominal effusions (*n* = 10). Some of the patients had more than one complication. The rest of the Fontan patients were considered uncomplicated (*n* = 17).

### 3.2 Near‐infrared fluorescence imaging

The average number of vessels analyzed per subject was 3.3 ± 1.2 (*n* = 48) and was not different between the two groups (*p* = 0.3460).

**FIGURE 2:**
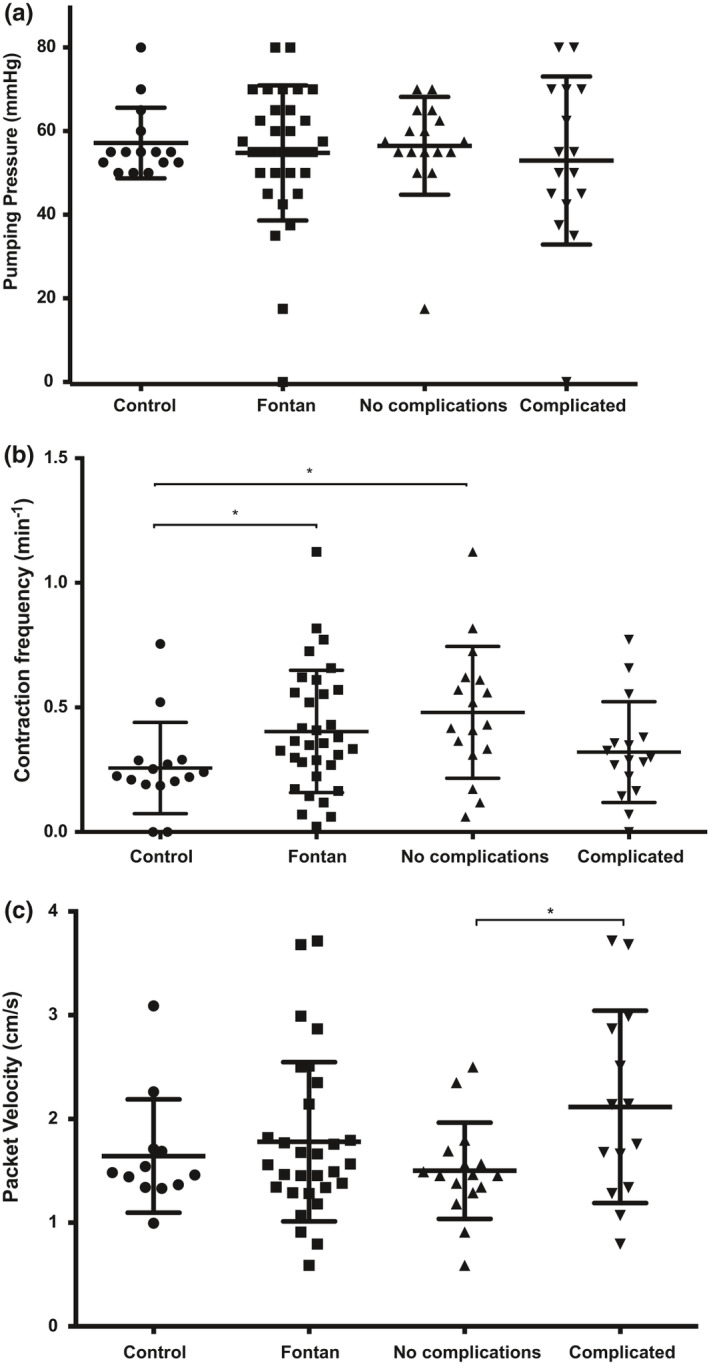
Dynamic baseline parameters estimated with NIRF imaging. (a) The average pumping pressure in patients with a Fontan circulation (*n* = 33) and subgroup divisions (not complicated and complicated) compared to healthy controls (*n* = 15) (Student's *t*‐test, *p* = 0.5921). (b) The average contraction frequency in patients with a Fontan circulation and subgroup divisions (not complicated [Student's *t*‐test, *p* = 0.0102*] and complicated [Student's *t*‐test, *p* = 0.3645]) compared to healthy controls (*p* = 0.0445*). (c) The average velocity in patients with a Fontan circulation and subgroup divisions (not complicated vs complicated, Student's *t*‐test, *p* = 0.0263*) compared to healthy controls (Student's *t*‐test, *p* = 0.5753). Abbreviations: Near‐infrared fluorescence (NIRF)

The pumping pressure in the lower leg lymphatics was not different between patients with a Fontan circulation, 54.8 ± 16.2 mm Hg, and healthy controls, 57.2 ± 8.4 mm Hg (*p* = 0.5921). No difference was found between the two subgroups or between the subgroups and controls (Figure 2a).

Baseline contraction frequency was higher in the Fontan group, 0.4 ± 0.3 min^−1^ compared to the controls, 0.3 ± 0.2 min^−1^ (*p* = 0.0445). The frequency among complicated Fontan patients showed no difference compared to controls (*p* = 0.3645), while uncomplicated patients showed an increase in contraction frequency (*p* = 0.0102), with contraction frequency being 0.3 ± 0.2 min^−1^ and 0.5 ± 0.3 min^−1^ respectively. There was no difference in frequency between the two subgroups of Fontan patients (*p* = 0.0621) (Figure 2b).

The velocity of lymph was not different between the Fontan patients (18 ± 8 mm×s^−1^) and the controls (16 ± 5 mm×s^−1^) (*p* = 0.5753). However, the complicated Fontan patients had a higher packet velocity (21 ± 9 mm × s^−1^) compared to the uncomplicated patients (15 ± 5 mm × s^−1^) (*p* = 0.0263) (Figure 2c).

No correlations were found between the baseline dynamic parameters.

### 3.3 Reserve capacity (Hyperthermia response)

**FIGURE 3:**
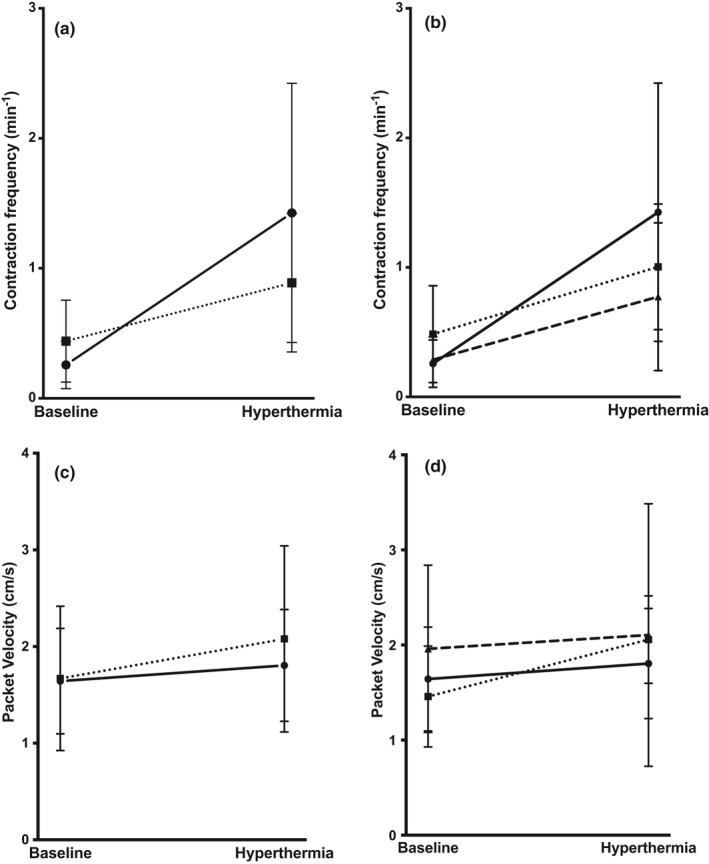
Dynamic parameters estimated with NIRF imaging after hyperthermia. (a) The contraction frequency increased significantly from baseline to after exposure of hyperthermia in both Fontan patients (*n* = 26, ■) (Student's *t*‐test, *p*<0.0001) and controls (*n* = 15, ●) (Student's *t*‐test, *p* = 0.0001). The increase in contraction frequency was lower in the Fontan patients compared to the controls (Student's *t*‐test, *p* = 0.0102). (b) No difference in response to hyperthermia was found between the uncomplicated Fontan patients (■) and the complicated Fontan patients (▲) (Student's *t*‐test, *p* = 0.8565). However, both the uncomplicated (*p* = 0.0441) and the complicated Fontan patients (Student's *t*‐test, *p* = 0.0356) had a lower reserve capacity compared to the healthy controls (●). (c) The packet velocity was unchanged after exposure to hyperthermia in both Fontan patients (■) (Student's *t*‐test, *p* = 0.5411) and controls (●) (Student's *t*‐test, *p* = 0.0824). (d) Subgroup analysis revealed that uncomplicated Fontan patients (■) increased packet velocity after hyperthermia (Student's *t*‐test, *p* = 0. 0.0291), while the complicated Fontan patients (▲) had unchanged packet velocity (Student's *t*‐test, *p* = 0.7378). No difference in response was found between the subgroups and controls (●). Abbreviations: Near‐infrared fluorescence (NIRF)

Compared to baseline, 5 min of hyperthermia lead to an increase in contraction frequency to 0.9 ± 0.5 min^−1^ (*p *< 0.0001) and 1.4 ± 1.0 min^−1^ (*p* = 0.0001) in Fontans and controls, respectively. The Fontan patients had a lower ability to increase contraction frequency (0.6 ± 0.5 min^−1^) compared to the healthy controls (1.2 ± 0.8 min^−1^) (*p* = 0.0102) (Figure 3a).

There was no difference in response to hyperthermia between the Fontan patients with no complication and those with complications (*p* = 0.8565). However, both groups had a lower reserve capacity compared to the healthy controls (uncomplicated, *p* = 0.0441; complicated, *p* = 0.0356) (Figure 3b).

Fontan patients with the lowest reserve capacity also had the lowest ability to generate lymphatic pressure (*p* = 0.0282). This association was not present for the healthy controls (*p* = 0.8102).

The velocity of the lymph remained unchanged after inducing hyperthermia for the controls (Figure 3c). However, Fontan patients with no complication could increase the velocity with 5 ± 7 mm × s^−1^ *(p* = 0.0291) after hyperthermia, while the complicated Fontan patients had unchanged velocity (*p* = 0.7378) (Figure 3d). No correlations were found between velocity and the other functional outcomes.

### 3.4 Lymphatic vessel morphology

**FIGURE 4:**
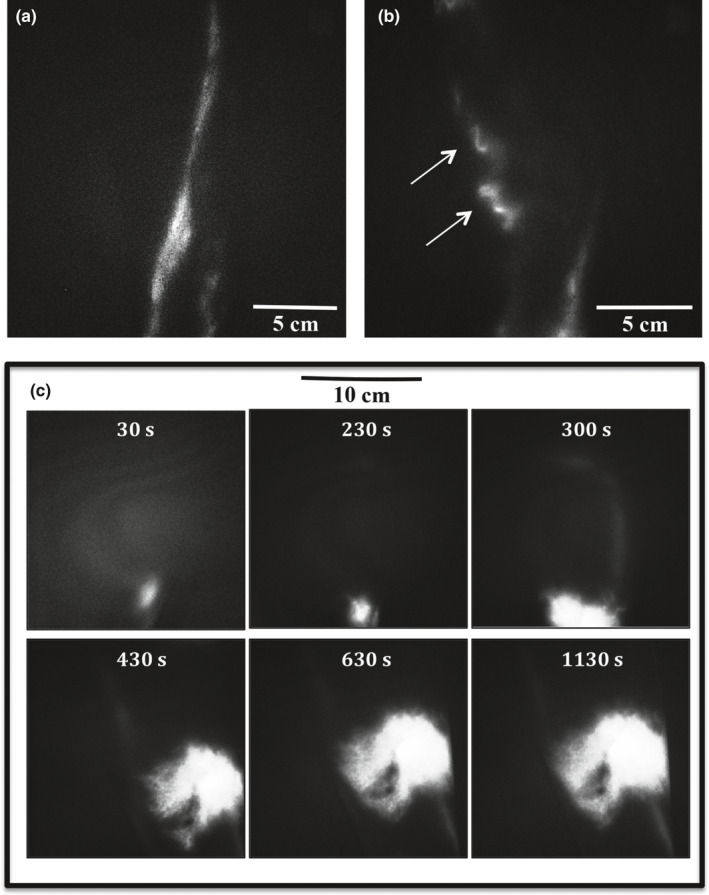
Morphological lymphatic abnormalities during NIRF imaging. (a) Still image of NIRF imaging sequence of morphological normal straight lymphatic vessel in the lower leg. (b) Still image of abnormal torturous lymphatic vessels in a Fontan patient (white arrows). c Example of dermal backflow in a Fontan patient after injection of ICG. The six still images represent time from injection of ICG. Four min after injection of ICG in the foot dermal backflow appears in the lower leg. Dermal backflow represents extravascular lymph leakage through dermal lymphatic collaterals, which is the end result of lymphatic obstruction. Over time the dermal backflow increases in size. Abbreviations: Indocyanine green (ICG). Near‐infrared fluorescence (NIRF)

Morphologically, eight Fontan patients (seven complicated) had abnormal lymphatic vessels in the lower leg. Five patients demonstrated tortuous vessels and three patients revealed areas with dermal backflow (backward leak of lymph; Figure 4). All three patients with dermal backflow were complicated; two patients were already diagnosed with PLE and one had lower leg edema. Only one control demonstrated a few tortuous vessels (Table 2).

### 3.5 Intraobserver reliability

Intraobserver ICC coefficient was 0.99 in pumping pressure estimation (*n* = 40) and for baseline contraction frequency ICC coefficient was 0.86 (*n* = 38). The baseline velocity measurement had an ICC coefficient of 0.75 (*n* = 25). After inducing hyperthermia, the contraction frequency measurements had an ICC coefficient of 0.95 (*n* = 34), while we found an ICC coefficient of 0.87 (*n* = 17) for package velocity.

### 3.6 Capillary filtration rate

All participants completed strain gauge plethysmography of the right calf, however the data from one Fontan patient was excluded due to artifacts hindering analysis.

**FIGURE 5:**
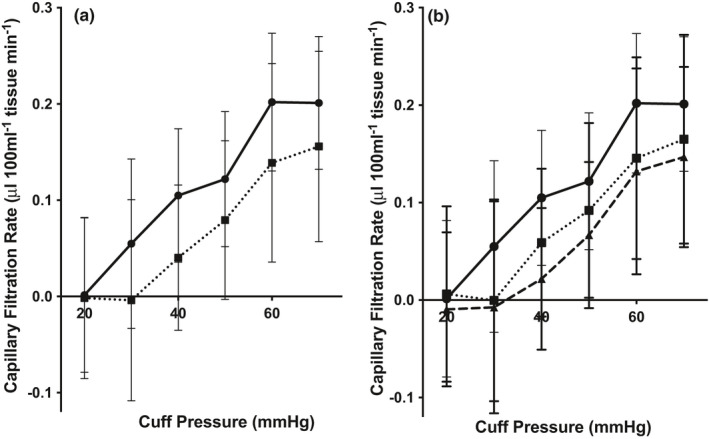
Capillary filtration rate, strain gauge plethysmography. (a) The capillary filtration rate of the lower leg was measured with strain gauge plethysmography at pre‐defined 10 mm Hg pressure steps (between 20–70 mm Hg). For each pressure from 30 mm Hg – 70 mm Hg the CFR for the Fontan patients (●, *n* = 32) was lower compared to the healthy controls (■, *n* = 15) (two‐way ANOVA; *p* = 0.0073). (b) The complicated Fontan patients (▲, *n* = 16) showed an even lower CFR at each pressure step compared to the healthy controls (■, *n* = 15) (two‐way ANOVA; *p* = 0.0027) than the uncomplicated Fontan patients (●, *n* = 16) (two‐way ANOVA; *p* = 0.0931). No difference in CFR was found between the two groups of Fontan patients (two‐way ANOVA; *p* = 0.2875) (Figure 3b)

CFR for Fontan patients was different from healthy controls (two‐way ANOVA; *p* = 0.0073). For each pressure from 30 to 70 mm Hg Fontan patients had a lower CFR (Figure 5a). The edema formation (increase in CFR) appeared at 42 ± 15 mm Hg in the Fontan patients while the increase already appeared at 30 ± 8 mm Hg for the controls (*p* = 0.0074) (Table 2). The complicated Fontan patients showed a higher difference in CFR compared to the healthy control (two‐way ANOVA; *p* = 0.0027) (Figure 5b) with increase in CFR starting at 48 ± 13 mm Hg. No difference was found between the two groups of Fontan patients (two‐way ANOVA; *p* = 0.2875) (Figure 5b). When comparing the eight patients with morphologically changed lymphatic vessels with the Fontan patients with normal lymphatic vessels, the onset of edema formation started at a higher venous occlusion pressure (54 ± 5 vs. 38 ± 3 mm Hg, Student's *t*‐test *p* = 0.0038). No correlations were found between onset of edema formation and lymphatic functional outcomes.

### 3.7 Periphery blood flow

The Fontan patients (*n* = 20, 1.7 ± 0.5 mL/min/100 mL tissue) had a lower PBF compared to the healthy controls (*n* = 8, 2.1 ± 0.2 mL/min/100 mL tissue), (*p* = 0.0088) (Table 2). No difference was found in PBF between the Fontan patients with no complications (*n* = 11, 1.6 ± 0.4 mL/min/100 mL tissue) and those complicated (*n* = 9, 1.7 ± 0.6 mL/min/100 mL tissue, *p* = 0.5865). No correlations were found between PBF and any of the NIRF imaging or CFR outcomes.

### 3.8 Norepinephrine

The Fontan patients (*n* = 25) had an increased concentration of NE at 378 ± 232 pg/mL compared to the control group (*n* = 11) at 228 ± 82 pg/mL (*p* = 0.0464) (Table 2).

The subgroup analysis revealed that the complicated Fontan patients (*n* = 12), had even higher levels of NE at 479 ± 268 pg/mL (*p* = 0.0073) compared to the control group, while the not complicated Fontan patients (*N* = 13) had NE levels at 285 ± 151 pg/mL not different to the control group (*p* = 0.2757). When comparing the two Fontan groups the complicated had increased levels of NE compared to the uncomplicated patients (*p* = 0.0344). Furthermore, levels of circulation NE were increased in the Fontan patients with morphological abnormalities compared to those without (*p *< 0.0001).

## 4 BNP

The Fontan patients (*n* = 25) had a concentration of BNP at 38 ± 39 pg/mL in contrast to the control group (*n* = 11) at 14 ± 19 pg/mL (*p* = 0.0634) (Table 2). The subgroup analysis showed no difference between the groups of Fontan patients with complications (*n* = 12) compared to the uncomplicated (*n* = 13) (*p* = 0.7100).

## 5 DISCUSSION

In this study, we revealed that peripheral lymphatic vessels in the Fontan patient contract 57% more frequently than healthy controls but when stressed by hyperthermia, the ability to increase contraction frequency is impaired. Thus, the lymphatic vessels in Fontan patients were unable to respond in a normal way, and only able to increase contraction frequency during stress twofold, compared to a fivefold increase amongst healthy controls. In accordance with previous findings in uncomplicated Fontan patients (Mohanakumar et al., 2019), the lymphatic contraction frequency was increased. In this study, we also looked at Fontan patients complicated with effusions and demonstrated that they in contrast had a lower contraction frequency during rest. At the microcirculation level the Fontan patients filtrated less fluid into interstitial space from the capillaries compared to the healthy controls at a dictated venous occlusion pressure and PBF was lower. Finally, NE blood levels were increased in the Fontan patients complicated with effusions and with morphologically changed lymphatic vessels. Echocardiography and levels of circulating BNP (Tang et al., 2003) ruled out that the fluid imbalance is caused directly by cardiac failure.

### 5.1 Impaired lymphatic drainage and not increased fluid filtration seems to be the cause of effusions in Fontan patients

Under normal conditions, the blood capillaries continuously filter fluid amounting to approximately 8 L daily that needs to be returned to maintain fluid balance (Levick & Michel, 2010). In 2010 Levick et al. proposed, after revising the fundamental principle governing fluid shifts laid down by Starling in 1896 (Starling, 1896), that downstream microvessels are not in a state of sustained fluid absorption and that tissue fluid balance depends critically on lymphatic drainage and not on venous reabsorption (Levick & Michel, 2010). This means that excess fluid accumulation is essentially caused by a mismatch between capillary filtration and lymphatic drainage.

Like the heart, lymphatic fluid transport is primarily a result of coordinated contractions and depends upon *contraction frequency* and *stroke volume* (Telinius et al., 2010). The coordinated contractions are a result of intrinsic contractions by the lymphatic vessels and not a central pump. This active pulsatile mechanism allows the lymphatic vessel to generate and regulate lymphatic flow against a hydrostatic pressure. Unidirectional valves divide the lymphatic vessels into functional contractile segments, named lymphangions (Breslin et al., 2018; Bridenbaugh et al., 2003; Telinius et al., 2010). The series of contractile lymphangions can be considered as an arrangement of small pumping chambers interconnected in chains terminating through the thoracic duct into the great veins of the neck (Breslin et al., 2018; Zawieja, 2009).

In the recent years, it has been repeatedly demonstrated that the lymphatic system is challenged in the Fontan circulation. Due to the high venous pressure, the afterload that the lymphatic vasculature has to work against is more than doubled in most Fontan patients (Biko et al., 2019; Dori et al., 2014a; Mohanakumar et al., 2019). To maintain fluid balance and avoid fluid effusion, a new “steady state” has to be established, so the lymphatic vasculature can remove the same amount of interstitial fluid back to the blood circulation, but against a higher afterload. We propose that the increased lymphatic contraction frequency in the uncomplicated Fontan patient is a response to the increased afterload/CVP. The chronotropic reaction to the increased CVP is supported by several studies on isolated lymphatic vessels in vitro. An acute elevation of afterload leads to combined increase in contraction frequency and inotropy (Breslin et al., 2018; Scallan et al., 2012). The low CFR rule out an increased preload. A low preload to the lymphatic vasculature in the Fontan patients has been shown earlier by *Krishnan et al*. They proposed that a higher filtration threshold may be an adaptive mechanism developed to chronic exposure to the high CVP, preventing onset of edema (Krishnan et al., 2009). However, as in heart failure, an increased resistance or afterload can be compensated initially, but when challenged beyond a certain point it can lead to long‐term failure and eventually effusions. This could explain why we see a difference in lymphatic pumping between the complicated and uncomplicated Fontan patients.

### 5.2 Morphological changes may be linked to the depressed functional reserve capacity

The structure of the lymphatic vasculature may explain the compensatory mechanism and the long‐term fatigue seen in the Fontan patients. As mentioned earlier, each lymphangion act as an independent contractile unit. Consequently, it is only affected by the state of the adjoining lymphangion attached upstream and downstream, respectively. Therefore, the afterload of 1 lymphangion will be the preload of the next downstream lymphangion (Breslin et al., 2018). Experiments on isolated series of several lymphangion showed that after an acute increase in afterload, the most distant upstream lymphangions pump significantly better than those lymphangions downstream and closest to the increased afterload. This suggests, that although there is an increase in CVP it is possible that the most peripheral lymphatic vessels are somewhat protected from the elevated afterload (Eisenhoffer et al., 1995). This serial protection in the periphery may explain why some lymphatic complications with effusions are long‐term complications and usually not seen shortly after completion of the Fontan circulation.

The loss of ability to maintain a healthy fluid equilibrium, seems to be counteracted by the plasticity of the lymphatic vasculature. The initial reaction to the Fontan circulation is dilatation (lymphangiectasia) or abnormal proliferation of collateral lymphatic vessels. The development of collaterals and dilatation of lymphatic vessels in Fontan patients with effusions has recently been demonstrated by MRI‐lymphangiography and was also seen in the current study, where seven out of eight Fontan patients with morphologically changed lymphatic vessels also were complicated with effusions (Biko et al., 2019; Dori et al., 2014a; Mohanakumar et al., 2019). Using dynamic ultrasound imaging, other studies have revealed a significant dilatation of the terminal part of thoracic duct in the neck of patients with a venous congestive condition (congestive heart failure, liver cirrhosis) (Seeger et al., 2009) and in Fontan patients (Sung et al., 2017). Other studies have experimentally investigated the thoracic duct drainage when systemic venous pressure is increased and have shown that when outflow pressure increases above thoracic duct pressure a linear decrease in lymphatic flow appears and at high outflow pressures drainage completely stops (Brace & Valenzuela, 1990). A recent study by Rossitto et al. addressed for the first time, the concern of a reduced lymphatic reserve in patients with acquired heart disease. They showed in skin biopsies from patients with heart failure but preserved ejection fraction that the peripheral lymphatic vessels exhibited structural and molecular alterations by dilatation of the lymphatic vessels and reduced expression of markers driving development and dynamic maintenance of lymphatic vessels and valves (Rossitto et al., 2020). Our results support these structural and molecular findings by adding that functional changes are also present in the peripheral lymphatics of patients with a heart condition.

Lymphatic valve incompetency has been suggested as a part of the cascade leading to impairment of lymphatic drainage. The bicuspid lymphatic valves are crucial for maintaining unidirectional flow and preventing backflow of lymph (Breslin et al., 2018).

Investigations have shown that the lymphatic valves tend to be open when there is no‐valve pressure gradient. Under normal condition, the outflow pressure exceeds the inflow pressure between contractions, so the outflow valves close, similar to the gating mechanism of the ventricular valves in the heart, and prevent backward flow (Davis et al., 2011). Moreover, the competency of valve closure is dependent of vessel diameter. When the vessel diameter is normal or small, like during systole, the trans‐valve pressure gradient is sufficient for valve closure, but when the vessel diameter reaches maximum, much higher transvalvular pressure gradients are needed for valve closure. This means that lymphatic tone is important for valve gating and in conditions were lymphatic vessels remain dilated, such as in lymphedema, valve malfunction may be present (Bertram et al., 2011; Breslin et al., 2018; Davis et al., 2011). In a condition where lymphatic valves remain open, fatigue of the lymphatic smooth cells in the vessel wall have been described and this would prevent forward drive of lymphatic fluid (Scallan et al., 2013). The increased outflow/afterload pressure combined with incompetent valves will be transferred to adjacent upstream lymphangions causing an overall failure of the lymphatic vasculature to move lymph forward and downstream (Scallan et al., 2013).

This phenomenon could be applied to the Fontan patients, where high CVP and valve incompetence, secondary to lymphatic dilatation (Biko et al., 2019; Dori et al., 2014a; Mohanakumar et al., 2019; Sung et al., 2017), endorses retrograde flow.

This could explain why Fontan patients complicated with effusion have lack of compensatory increase in contraction frequency during rest and why we find a correlation between a lower reserve capacity and a decreased ability to generate lymphatic pressure.

Increased velocity of flow has previously been reported in the extremities of patients with lymphedema (Tan et al., 2011). Interestingly, increased propulsion velocity was also found in the current study in the patients complicated with effusions. At this point an explanation for this finding would be entirely speculative and further studies have to be performed to address this finding.

### 5.3 Increased norepinephrine could be a result of distorted fluid equilibrium

Ex vivo studies of human lymphatic vessels showed that alpha‐adrenergic stimulation with NE as well as stimulation of the adrenergic nerves increases contraction frequency (Mohanakumar et al., 2018; Telinius et al., ,^2010^, ^2014^). The increased levels of NE could be speculated to be a possible pathway to how the Fontan patients increase lymphatic contraction frequency. However, our in vivo results could not show the direct association between levels of NE and a higher contraction frequency in the superficial lymphatics. Others, have already described that neurohumoral activation is present in the Fontan patients with higher levels of circulating NE (Inai et al., 2005; Turquetto et al., 2018).

Increased level of circulating NE is also seen in patients with congestive heart failure, thought to increase as a result of low blood pressure and to increase peripheral vascular resistance (Inai et al., 2005). Although most of the Fontan patients in these and the current study were in NYHA I and had no severe ventricular systolic dysfunction shown with echocardiography, the elevated NE could be explained as an adaptive response to maintain sufficient blood flow after establishing the Fontan circulation and the possible positive effect on the lymphatic contractility a favorable consequence. But interestingly, the NE levels was only significantly elevated in the Fontan patients complicated with effusions and with lymphatic morphological changes compared to the controls. Perhaps, NE could be a potential biomarker for the pathophysiology that pushes to the transition from uncomplicated to a complicated Fontan patient. Further studies should be conducted to precisely investigate the association between the Fontan circulation, lymphatic dysfunction and levels of circulating NE.

### 5.4 Limitations

The CVP was not measured at the time of investigation due to the invasive nature of the procedure and earlier measurements were not available. At this point we are, therefore, not able to correlate our findings directly with the CVP at the time of examination. All Fontan circulations are, however, known to function on the basis of an elevated CVP (Biko et al., 2019; Dori et al., 2014a; Mohanakumar et al., 2019). To know the patency of the femoral vein would have been valuable, although no clinical signs suggested venous obstructions.

The introduction of NIRF imaging has allowed for dynamic functional investigation of the lymphatic vasculature. Unfortunately, the ICG‐based imaging technique only allows for visualization of the superficial peripheral lymphatic vessels within a depth of 1–2 cm into the tissue. Taking into account that the regional variation of the lymphatic function has been reported (Gashev et al., 2004; Groenlund et al., 2017; Kelly et al., 2020b), care must be taken extrapolating the results from the peripheral lymphatic vessels to the larger central lymphatic vessels. However, multiple studies report universal morphological changes (Biko et al., 2019; Dori et al., 2014a; Mohanakumar et al., 2019). Hence, we consider it reasonable to expect that the functional lymphatic change is a universal decay.

### 5.5 Clinical importance

The functional capacity of the lymphatic vasculature plays a role for both the early adaptation after the Fontan circulation and the late‐onset effusion experienced by the Fontan patients. Our results support the notion that the Fontan patients have at least two different adaptive capacity responses of the lymphatic vasculature. Innate lymphatic anomalies leading to leak of lymph in the early phase post‐Fontan (Biko et al., 2019; Dori et al., 2014a) and effusions after several years with a Fontan circulation, like the patients in the current study (Mohanakumar et al., 2019). In the patients who succumb to late‐onset complication with effusions, the long‐term exposure to an inherent high CVP combined with the nature of plasticity seen in the lymphatic vasculature and a lower reserve functional capacity could be the triad of mechanisms leading to impaired lymphatic drainage, fluid accumulation or effusions, culminating with severe end‐organ failure. The question is whether impaired functional reserve capacity in these Fontan patients is a consequence of the long‐term exposure to a chronic elevated CVP resulting in exhaustion or an innate underdevelopment of the lymphatic vasculature. Lymphatic functional investigation, using novel techniques such as NIRF imaging, on patients before and immediately after completion of the Fontan circulation could get us closer to an answer.

In this lymphatic imaging study of Fontan patients with and without effusions, we demonstrate that the capacity to increase lymphatic function during fluid‐stress is impaired in both groups. The study underlines the need for functional lymphatic studies, pre and post‐Fontan, to uncover if the lymphatic dysfunction is inherent or caused by an exhaustion after many years of exposure to a high CVP.

## CONFLICT OF INTEREST

None.

## Author Contributions

SM, BK, AT, FA, PM, MJ, and VH was part of designing and initiating the study. AT, LA, MB, JS, FA, PM, and MJ recruited study participant and handled all the logistical and practical aspect of the study. SM, BK, VH, AT, MA, LA, MB, JS, and PM contributed in data collection, data analysis, and reviewing the medical records. All the authors contributed in drafting and reviewing the manuscript upon submission.

## Supporting information




Movie 1

Movie 2
